# Advanced Interatrial Block across the Spectrum of Renal Function

**DOI:** 10.3390/medicina60061001

**Published:** 2024-06-18

**Authors:** Marco Marano, Luigi Senigalliesi, Rossella Cocola, Mariarosaria Fontana, Erika Parente, Vincenzo Russo

**Affiliations:** 1 Unit of Nephrology and Dialysis, Maria Rosaria Clinic, Via Colle San Bartolomeo, 80045 Pompei, Italy; marano965@gmail.com (M.M.); luseniga@gmail.com (L.S.); rossella.cocola@gmail.com (R.C.); mariarosaria.fontana@hotmail.it (M.F.); 2 Cardiology Unit, Department of Medical Translational Sciences, University of Campania “Luigi Vanvitelli”, Monaldi Hospital, Via Leonardo Bianchi, 80126 Naples, Italy; parente-erika@libero.it

**Keywords:** advanced interatrial block, atypical patterns, chronic kidney disease, dialysis, P-wave

## Abstract

*Background and Objective*: Interatrial block (IAB) is defined as a conduction delay between the right and left atria. No data are available about the prevalence of both partial IAB and advanced IAB among the different stages of chronic kidney disease. The aim of this study was to describe the prevalence and type of advanced IAB across the spectrum of renal function, including patients on dialysis and the clinical characteristics associated with advanced IAB. *Materials and Methods*: Retrospective, single-center study of 151 patients consecutively admitted to the Nephrology and Ophthalmology Unit for 3 months. The study population was divided into three groups according to stages of chronic kidney disease. We evaluated the prevalence and pattern of IAB among the groups and the clinical characteristics associated with advanced IAB. *Results*: The prevalence of partial IAB was significantly lower in end-stage kidney disease (ESKD) group compared to control group (36.7% vs. 59.6%; *p* = 0.02); in contrast the prevalence of advanced IAB was significantly higher in both chronic kidney disease (CKD) (17.8% vs. 5.3%, *p* = 0.04) and ESKD group (24.5% vs. 5.3%, *p* = 0.005) compared to control group. The atypical pattern of advanced IAB was more frequent in both the ESKD and CKD group than in the control group (100% and 75% vs. 33.3%; *p* = 0.02). Overall, among patients that showed advanced IAB, 17 (73.9%) showed an atypical pattern by morphology and 2 (8.7%) showed an atypical pattern by duration of advanced IAB. The ESKD group was younger than the control group (65.7 *±* 12.3 years vs. 71.3 ± 9.9 years; *p* = 0.01) and showed a higher prevalence of beta blockers (42.9% vs. 19.3%; *p* = 0.009), as in the CKD group (37.8% vs. 19.3%; *p*= 0.04). *Conclusions:* The progressive worsening of renal function was associated with an increasing prevalence of advanced IAB. Advanced IAB may be a sign of uremic cardiomyopathy and may suggest further evaluation with long-term follow-up to investigate its prognostic significance in chronic kidney disease.

## 1. Introduction

Interatrial block (IAB) is defined as a delay of conduction between the right and left atria due to impulse delay or blockage resulting in prolonged P-wave duration with (advanced IAB) or without (partial IAB) biphasic morphology in the electrocardiographic inferior leads [1,2]. The pathological substrate of IAB is characterized by the fibrotic substitution of normal atrial musculature due to intracellular destruction and replacement with glycogen and collagen deposition between cells, the collagen deposition disrupting the normal atrial current flow [3]. In two population-based studies [4,5], including 14,625 and 152,759 patients, 0.5% of the study population, respectively, 45–64 and 50–90 years old, had advanced interatrial block (A-IAB) at baseline. Among 202 patients aged around 61–103 years (mean age 81.75 years), the prevalence of advanced IAB was 6% [6], and the prevalence was even higher in 80 subjects older than 100 years in the study of Martínez-Sellés et al. [6]. The prevalence of IAB increases with age [7,8], and it has been demonstrated to be a predictor of atrial fibrillation [4,9,10,11,12,13,14]; patients aged > 60 years with A-IAB have a 50% risk for developing atrial fibrillation (AF) at 6 years; meanwhile, the risk for AF is only 10% among patients who do not have atrial fibrillation [2]. Among patients with chronic kidney disease (CKD), the prevalence of IAB was high; in particular, Solak et al. [15] showed a prevalence of both partial and A-IAB in end-stage kidney disease (ESKD), respectively, 47% and 14%, but they excluded from their analysis diabetic patients and patients with multiple cardiovascular diseases, and the definition of IAB adopted did not include the evaluation of the criteria established by Bayés de Luna et al. [16]. For these reasons, the diagnosis of IAB is sometimes misunderstood or underestimated according to the current definitions.

Among hemodialysis patients, the prevalence of partial and advanced IAB was 49.3% and 7%, respectively [17]. It has also been suggested that IAB is an independent risk factor for stroke [18,19,20], cognitive impairment [21,22,23], dementia [21], atrial cardiomyopathy such as a rare presentation of a laminopathy [24], thrombotic events, acute respiratory distress syndrome (ARDS) in need of intubation [25] and cardiovascular mortality in several clinical settings [26,27]. Advanced IAB has been described as a sign of uremic cardiomyopathy among patients with chronic kidney disease (CKD) in hemodialysis [17,18,19,20,21,22,23,24,25,26,27,28]; however, no data are available on the prevalence of both partial and advanced IAB among different stages of CKD. The aim of this study was to describe the prevalence and type of advanced IAB across the spectrum of renal function.

## 2. Materials and Methods

### 2.1. Study Design

We retrospectively evaluated 186 consecutive patients admitted to Nephrology and Ophthalmology Unit of Maria Rosaria Clinic, Pompeii, Italy from 1 May and 30 July 2022. The medical history, physical examination, electrocardiogram (ECG), echocardiography, laboratory data, and pharmacological therapy were collected. Glomerular filtration rate (GFR) was estimated by CKD-EPI equations based on serum creatinine, gender, and age. Patients with no sinus rhythm (n = 23) and poor quality of ECG (n = 12) were excluded. The study population was divided into three groups: the first group was called “Control (CTRL) group”, which included patients with GFR ≥ 60 mL/min/1.73 m^2^; the second group was called “Chronic kidney disease (CKD) group”, which included patients with GFR < 60 mL/min/1.73 m^2^ but not on dialysis; the third group was called “End-stage kidney disease (ESKD) group”, which included patients on dialysis. All patients with end-stage kidney disease (ESKD) underwent hemodialysis three times per week, and each session lasts about four hours. We evaluated the prevalence and pattern of advanced IAB among groups and the clinical characteristics associated with advanced IAB. This study was conducted according to the Declaration of Helsinki; written informed consent for data collection was obtained from the patients; the local ethical committed approved the analysis.

### 2.2. Electrocardiographic Measurements

All subjects underwent a routine standard 12-lead surface ECG recorded at a paper speed of 50 mm/s and gain of 10 mm/mV in the supine position and were breathing freely but not allowed to speak during the ECG recording. To avoid diurnal variations, we generally took the ECG recordings at the same time (9:00–10:00 A.M.). The analysis was performed by one investigator only, without knowledge of subject’s clinical status. ECGs were transferred to a personal computer by an optical scanner and then magnified 400 times by Adobe Photoshop software 22.x (Adobe Systems Inc., San Jose, CA, USA). P-wave duration measurement was manually performed with the use of computer software (Configurable Measurement System, Windows 10). Intra-observer coefficients of variation for P-wave variables were found to be less than 5% and not significant. In each electrocardiogram lead, the analysis included three consecutive heart cycles wherever possible. ECG with measurable P-wave in less than ten leads were excluded from analysis. The onset of P-wave was defined as the junction between the isoelectric line and the start of P-wave deflection; the offset of the P-wave was defined as the junction between the end of the P-wave deflection and the isoelectric line. If starting and endpoints were not clear, the derivations including these points were taken as excluding criteria from the study. Maximum and minimum P-wave durations were measured. Maximum P-wave duration was defined as the longest P-wave duration, and minimum P-wave duration was defined as the shortest P-wave duration. 

IAB was defined according to new criteria established by Bayés de Luna et al. [16]; in particular, partial IAB was defined by P-wave duration ≥ 120 ms. Advanced IAB was defined by P-wave duration ≥ 120 ms and P-wave morphology biphasic [±] in II, III, and aVF leads (typical pattern) or at least the last part of P-wave being negative in lead aVF (atypical by morphology) as a consequence of left atrium activation retrogradely from the AV zone, as a result of complete block of sinus impulse in some part of the Bachmann bundle. This specific activation pattern is the hallmark of advanced IAB. The [±] morphology in II, III, and aVF leads may also occur if P-wave duration < 120 ms, as in the case of atypical pattern by duration of advanced IAB.

### 2.3. Echocardiographic Measurements

All subjects underwent two-dimensional transthoracic echocardiography (TTE) according to standard technique [29]. 

The left atrial (LA) size was measured using linear dimension of antero-posterior (AP) diameter [30,31], in the parasternal long-axis view perpendicular to the aortic root long axis, and measured at the level of the aortic sinuses by using the leading-edge-to-leading-edge convention. An AP diameter of, respectively, 27–38 mm and 30–40 mm was considered normal range in male and female [32,33].

The LV ejection fraction (EF) was measured using the biplane method of disks (modified Simpson’s rule), calculated with the following formula: EF = (end diastolic volume (EDV)—end systolic volume (ESV))/EDV. Left ventricular ejection fraction (LVEF) ranged from, respectively, 52–72% and 54–74% was considered within the normal range in males and females [29]. Aortic regurgitation (AR) was evaluated considering qualitative, semi-quantitative, and quantitative parameters [34,35] such as color jet-proximal flow convergence, vena contracta (VC), jet width, aortic effective regurgitant orifice area [EROA] by the PISA method, and pressure half-time (PHT), defined as mild, moderate and severe.

Mitral regurgitation (MR) was evaluated considering qualitative, semi-quantitative, and quantitative parameters [34,36] such jet-proximal flow convergence, VC, mitral EROA by the PISA method that was defined as mild, moderate, and severe.

### 2.4. Interatrial Block

The interatrial block, by analogy with other types of blocks, may be of first (partial), second (transient, atrial aberrancy), or third degree (advanced). Partial IAB is defined as a delayed transmission of the sinus impulse through the Bachmann’s region; the ECG shows a P-wave duration ≥ 120 ms with normal morphology. Advanced IAB is defined as a block of the sinus impulse in the Bachmann’s region, and as the rest of the septum is predominantly connective tissue, the breakthrough to left atrial was achieved retrogradely from the AV junction zone or other near zone [16,37,38]; the ECG shows a P-wave duration ≥ 120 ms with biphasic morphology in the inferior leads. This latter activation is a cause of left atria electromechanical dysfunction and left atrio-ventricular dyssynchrony and has been associated with left ventricular diastolic dysfunction [38].

### 2.5. Statistical Analysis

The distribution of continuous data was tested with Kolmogorov–Smirnov and the Shapiro–Wilk test. Normally distributed variables were expressed as mean ± standard deviation (SD). Non-normally distributed variables were expressed as median and interquartile range (IQR). Categorical variables were reported as numbers and percentages. Continuous normally distributed variables were compared by using Student’s *t*-test. Categorical variables were compared with the χ^2^ test; the Yates correction for continuity was used if expected frequencies were less than 5 in 2 × 2 χ^2^ tables. The association between IAB and the baseline clinical characteristics was evaluated using logistic regression models and presented as odds ratio (OR) with 95% confidence intervals (CI). All variables showing a *p* value < 0.05 at univariable analysis were tested in the multivariable model. For all tests, a *p* value < 0.05 was considered statistically significant. All statistical analyses were performed using STATA software, version 11.1 SE (Statacor, College Station, TX, USA: StataCorp LLC).

## 3. Results

A total of 151 patients (70.4 ± 11.3 years. 53.6% males) were included in the study. The baseline characteristics of the study population are summarized in Table 1. The ESKD group was younger than the control group (65.7 *±* 12.3 vs. 71.3 ± 9.9; *p* = 0.01) and showed a higher prevalence of betablockers (42.9% vs. 19.3%; *p* = 0.009), as in the CKD group (37.8% vs. 19.3%; *p* = 0.04). The CKD group showed a higher LA diameter than that of the control group (40.5 ± 1.6 mm vs. 39 ± 3.1 mm, *p* = 0.004). The ESKD group showed reduced LVEF compared to control group (54 ± 5% vs. 60.3 ± 2.7%; *p* < 0.0001).

**Figure 1:**
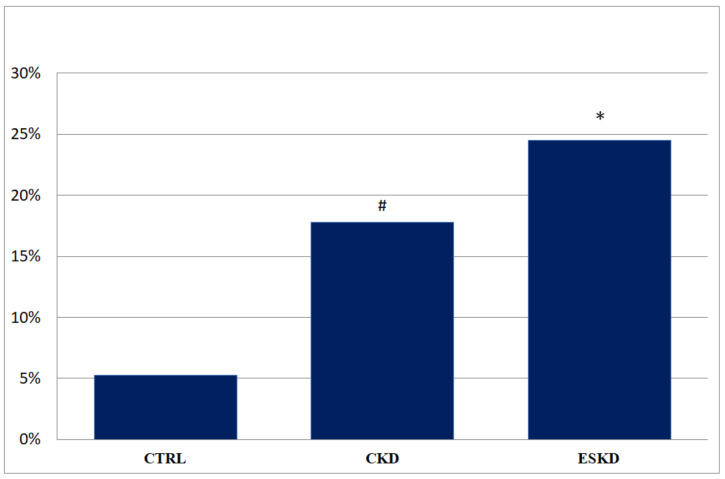
Prevalence of advanced IAB among study population. CTRL: control group; CKD: chronic kidney disease; ESKD: end-stage kidney disease (**#**: *p* = 0.04; *: *p* = 0.005).

The prevalence of partial IAB was significantly lower in the ESKD group compared to the control the group (36.7% vs. 59.6%; *p* = 0.02); in contrast, the prevalence of advanced IAB was significantly higher in both the CKD (17.8% vs. 5.3%, *p* = 0.04) and ESKD group (24.5% vs. 5.3%, *p* = 0.005) compared to control group. The prevalence of advanced IAB among the study population is shown in Figure 1.

In the univariable analysis, ESKD was the only clinical variable correlated to the presence of advanced IAB (OR: 2.68; 95% CI: 0.09–6.62; *p* = 0.03). Conversely, GFR ≥ 60 mL/min/1.73 m^2^ (OR: 0.20; 95% CI: 0.06–0.72; *p* = 0.01) was independently associated with advanced IAB. The univariable regression logistic analysis for clinical characteristics associated with advanced IAB is shown in Table 2.

**Figure 2:**
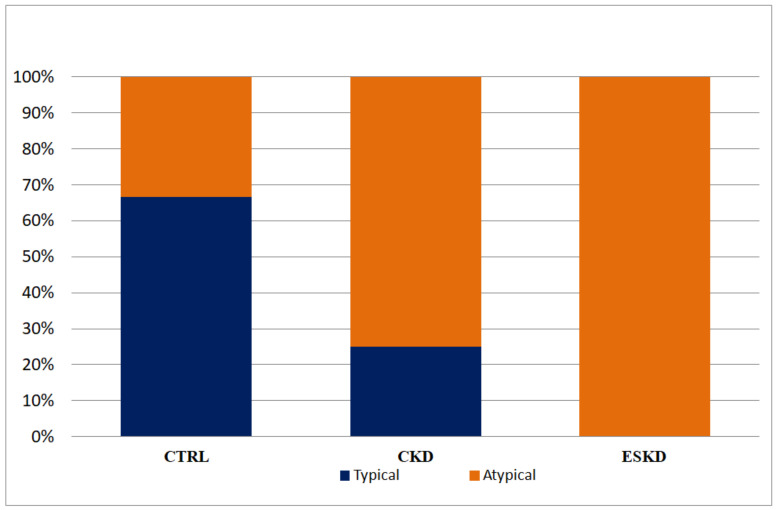
Patterns of advanced IAB (typical or atypical) across study groups. CTRL: control group; CKD: chronic kidney disease; ESKD: end-stage kidney disease (*p* = 0.02).

The atypical pattern of advanced IAB was more frequent in both the ESKD and CKD groups than in the control group (100% and 75% vs. 33.3%; *p* = 0.02). Among patients that showed advanced IAB, 17 (73.9%) showed an atypical pattern by morphology and two (8.7%) showed an atypical pattern by duration of advanced IAB. The prevalence of typical and atypical pattern of advance IAB across different groups is shown in Figure 2.

**Figure 3:**
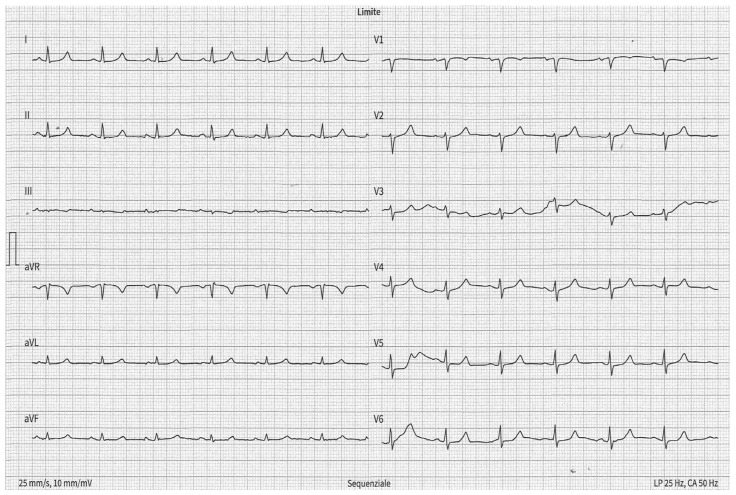
Partial IAB. The ECG shows P-wave duration = 120 ms in the inferior leads.

**Figure 4:**
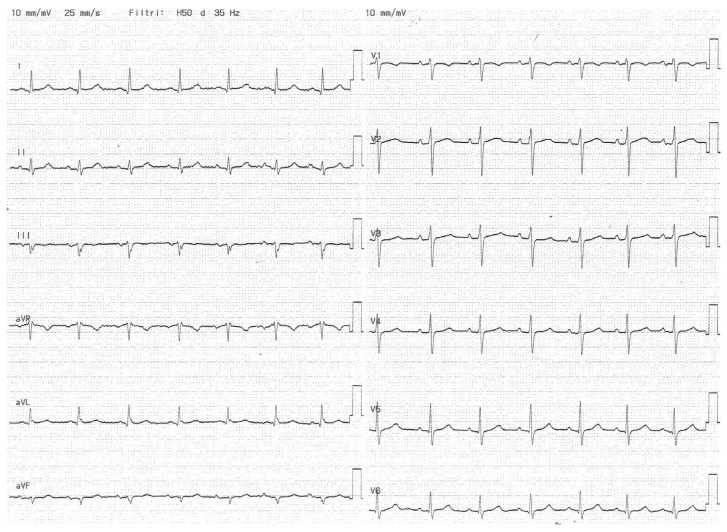
Advanced IAB. The ECG shows P-wave duration > 120 ms and biphasic morphology in the inferior leads.

**Figure 5:**
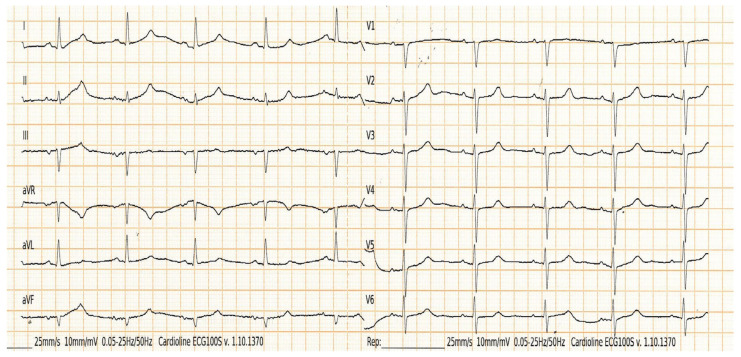
Atypical pattern of advanced IAB by morphology.

Figure 3 and Figure 4 show partial IAB and advanced IAB, respectively; Figure 5 shows atypical pattern of advanced IAB by morphology.

## 4. Discussion

### 4.1. Relationship between Chronic Kidney Disease and Electrocardiographic Abnormalities

CKD was defined as the presence of kidney damage or an eGFR less than 60 mL/min/1.73 mt^2^, at least for three months, irrespective of the causes [39]. It was a progressive loss of kidney function that could result in renal replacement therapy (dialysis or transplantation). The prevalence of CKD accounts for 10% to 14% in the general population [40]. Among CKD patients, compared with the general population, cardiovascular disease (CVD) was more frequent and severe [41]. CKD was associated with several CVD outcomes, including coronary heart disease, stroke, peripheral artery disease, arrhythmias, heart failure, and venous thrombosis [42]. Impaired glomerular filtration rate increases the risk for cardiovascular events, morbidity, and mortality, so understanding the status of CKD and preventing progression of chronic kidney disease is crucial to prevent cardiovascular disease [41,42]. This association could be referred to as endothelial cell dysfunction, which is found in micro- or macroalbuminuria; this dysfunction is defined as one of the principal pathophysiological mechanisms associated with cardiovascular risk factors. In addition, diabetes, obesity, and hypertension lead to the activation of the renin–angiotensin system, oxidative stress, elevated asymmetric dimethylarginine (ADMA), low-grade inflammation with increased circulating cytokines, and dyslipidemia, which are pathophysiological mechanisms that play a role in the association of renal failure and CVD [43]. ECG remains an essential tool for the early evaluation of cardiac rhythm abnormalities which may reflect the myocardium status or may be a consequence of ionic current dysregulation, metabolic changes, serum electrolytes alterations, and secondary effects of medications [37]. Electrocardiographic abnormalities are common in CKD patients and predict future cardiovascular events [44,45]. The link between impaired renal function and cardiac rhythm abnormalities has been explained by the blood electrolyte disturbances resulting from the dysregulation of electrolyte handling in the renal tubules and cardiac remodeling due to circulating uremic toxins, systemic inflammation, and sympathetic overactivity [46]. The relationship between CKD and AF is bidirectional since CKD increases incidence of atrial fibrillation and AF increases CKD progression [47]. Sudden cardiac death (SCD) represents approximately 22% of all deaths in ESKD patients [48,49]. Among them, an important clinical entity is the BRASH syndrome (bradycardia, renal failure, AV nodal blockade, shock, and hyperkalemia) characterized by the synergistic effects of AV nodal blockers and renal impairment that lead to severe bradycardia and hyperkalemia [50,51].

Several studies showed a QT interval prolongation following the impairment of renal function, especially in CKD patients in need of hemodialysis [49,50,51], becoming the more common ECG finding in ESKD [44]. This phenomenon is caused by increased inward current (sodium or calcium channels) or a decreased potassium outward current (potassium channel), which was found to characterize the electrolyte acid–base balance disorder in ESKD patients [52,53]. Among CKD patients on hemodialysis, a heterogeneity of regional ventricular repolarization leading to increased QTc interval dispersion has been shown [54]. 

Previous studies evaluated the prognostic role of electrocardiographic parameters among CKD patients. Flueckiger et al. [55] reported that the prolongation of ECGs interval (PR, QRS, or QTc) were associated with higher mortality. One (HR: 1.69) and two or more (HR: 2.58) ECGs interval prolongation were independent predictors of death among 930 patients with ESKD in need of kidney transplantation

Dobre et al. [56] showed that both major (ventricular conduction defect, Q-wave abnormalities, isolated ST-T–wave abnormalities, first-degree atrioventricular block, left ventricular hypertrophy) and minor (minor isolated ST-T–wave abnormalities, long QT interval, incomplete right bundle branch block) ECG abnormalities were independent predictors of cardiovascular events (HR: 2.15 and 1.24, respectively) and cardiovascular death (HR: 2.27 and 1.48, respectively) in CKD patients.

In this study, among 5888 patients, 1192 (20.2%) had CKD at baseline on different stages (CKD stage 3, 4, 5); during the mean follow-up of 10.3 ± 3.8 years, 814 (68.3%) participants died; of these deaths, primary arrhythmia represented 8.2%, and secondary arrhythmic mechanism represented 33.9%.

De Castroviejo et al. [57] analyzed the prevalence of bundle brunch block (BBB) in 211 patients starting hemodialysis or peritoneal dialysis for the first time; BBB was a factor indicative of a poor prognosis for higher rates of mortality and major cardiovascular events, particularly when the conduction defect was a left BBB. No studies have shown a relationship between the progressive worsening of renal function and the prevalence of interventricular conduction defects.

### 4.2. Interatrial Block and Left Atrial Enlargement

Left atria enlargement (LAE) has been associated with adverse cardiovascular events, including AF, stroke, and mortality. Even if P wave duration and IAB are associated with LAE, an electrocardiogram cannot be used as the sole diagnostic test to assess LAE, due to its low sensitivity and specificity [58,59], so interatrial blocks and atrial enlargement were defined as separate entities [60]. Advanced IAB was not independently associated with LAE. IAB shows a P wave of 120 milliseconds or more, usually bimodal, especially visible in leads I, II, or III, and in the absence of left atrial enlargement (LAE), the P wave morphology in V1 presents a P wave negative mode that is less evident than in cases of LAE, because in the absence of LAE, the P loop is directed in a less backward direction. In fact, it has been considered that the wide and bimodal P wave of LAE is better explained because of underlying IAB rather than the longer distance that the impulse has to travel [61,62,63,64].

### 4.3. Main Findings 

The main findings of our study are the following: the prevalence of advanced IAB increases across the spectrum of renal function, ranging from 17.8% in patients with CKD, defined as GFR < 60 mL/min/1.73 m^2^, to 24.5% among those on dialysis. Atypical advanced IAB, mostly by morphology, was the most frequent pattern among patients with renal dysfunction. Advanced IAB was not independently associated with LAE.

The occurrence of IAB was reported in 31–60% of patients with severe kidney disease [15,17,65] and up to 40% of patients on dialysis for a long time [66]; no data on the prevalence have been reported among patients with early-stage kidney disease.

Previous studies showed that the prevalence of advanced IAB was about 9.3% among dialysis patients and it was associated with atrial arrhythmias and other clinical outcomes [17]. The prevalence of advanced IAB among our patients on dialysis was two-fold higher; moreover, advanced IAB was recorded in 5% of patients with GFR > 60 mL/min and in 18% of patients with GFR < 60 mL/min/1.73 m^2^. Our data suggest that the electrical atrial impairment progressively increases as kidney disease worsens, supporting the hypothesis that advanced IAB might be the electrocardiographic sign of the atrial uremic cardiomyopathy [28]; however, the serum levels of uremic toxins were not measured, so it was not possible to demonstrate this association among our study population. As regards the relationship between dialysis treatment and advanced IAB, it may be explained by its harmful effect on atrial conduction velocity [66,67], leading to the worsening of IAB (from partial to advanced) [68] or the onset of atrial fibrillation, called Bayés syndrome [16]. Among our study population, no patients showed second-degree IAB (intermittent), confirming the rarity of this condition; only two cases were previously reported in dialysis patients [61,62,63,64,65]. Actual diagnostic criteria, including atypical pattern of advanced IAB, have redrawn the relationship between advanced IAB and kidney disease. More than 80% of instances belong to the atypical pattern, almost exclusively atypical by morphology.

### 4.4. Clinical Perspectives

Despite the emerging technologies, ECG remains an easy, non-operator-dependent, inexpensive, and non-invasive tool for the early identification of cardiac rhythm disorders.

The diagnosis of IAB may be an additional and easy marker for risk stratification of adverse cardiovascular events of patients across the spectrum of renal function. In particular, the occurrence of advanced IAB in CKD patients may stratify those at increased risk of arrhythmias and in need of more careful evaluation, improving the diagnostic and therapeutic management of patients with CKD.

### 4.5. Limitation

Our results should be interpreted while considering the limitations related to the study’s retrospective, observational, and single-center nature. The small sample size of the study population may have reduced the statistical power of the results. Further studies should be performed to confirm our preliminary findings. In the presence of P-IAB, the LAE is relatively common; it has been previously considered that the wide and bimodal P-wave of LAE is better explained because of underlying IAB rather than the longer distance that the impulse has to travel [66,67,68]. However, this is the first study to evaluate the prevalence of advanced IAB across the spectrum of renal function.

## 5. Conclusions

The progressive worsening of renal function was associated with an increasing prevalence of advanced IAB. These data support the hypothesis that advanced IAB, in particular that which is atypical by morphology, may be a sign of uremic cardiomyopathy. Further evaluations are needed to investigate the prognostic significance of advanced IAB across the spectrum of renal function.

## Figures and Tables

**Table 1:** Baseline characteristics of study population.

	Control Group n (57)	CKD Group n (45)	ESKD Group n (49)	*p* (CKD vs. Control)	*p* (ESKD vs. Control)
Male gender, n (%)	31 (54.4)	21 (46.7)	30 (61.2)	0.4	0.5
Age, mean ± SD, years	71.3 ± 9.9	74.5 ± 10.4	65.7 *±* 12.3	0.11	0.01
Diabetes mellitus, n (%)	11 (19.3%)	18 (40%)	15 (30.6%)	0.4	0.2
Hypertension, n (%)	32 (56.1%)	29 (64.4%)	25 (51%)	0.4	0.6
CAD, n (%)	10 (17.5%)	7 (15.6%)	13 (26.5%)	0.8	0.3
AF, n (%)	1 (1.8%)	2 (4.4%)	1 (2%)	0.4	0.9
LA diameter, n (%)	39 ± 3.1	40.5 ± 1.6	39.2 ± 3.3	0.004	0.75
LVEF (%), mean ± SD	60.3 ± 2.7	60 ± 1.4	54 ± 5	0.5	<0.0001
Mild AR, n (%)	4 (7.1)	0	1 (2)	0.07	0.22
Mild MR, n(%)	4(7.1)	2 (4.4)	5 (10.2)	0.57	0.57
1st-degree AV block, n (%)	2 (3.5%)	6 (13.3%)	4 (8.2%)	0.07	0.3
RBBB, n (%)	6 (10.5%)	3 (6.7%)	3 (6.1%)	0.5	0.4
LBBB, n (%)	0 (0%)	0 (0%)	1 (2%)	/	/
LAFB, n (%)	6 (10.5%)	2 (4.4%)	7 (14.3%)	0.3	0.5
Partial IAB, n (%)	34 (59.6%)	20 (44.4%)	18 (36.7%)	0.1	0.02
Advanced IAB, n (%)	3 (5.3%)	8 (17.8%)	12 (24.5%)	0.04	0.005
Oral Anticoagulant Drugs, n (%)	4 (7%)	3 (6.7%)	1 (2%)	0.9	0.2
ACE-I/ARB, n (%)	22 (38.6%)	24 (53.3%)	11 (22.4%)	0.1	0.07
β-blockers, n (%)	11 (19.3%)	17 (37.8%)	21 (42.9%)	0.04	0.009
Antiarrhythmic drugs, n (%)	2 (3.5%)	3 (6.7%)	4 (8.2%)	0.5	0.3

CAD: coronary artery disease; AF: atrial fibrillation; LA: left atria; LVEF: left ventricular ejection fraction; AR: aortic regurgitation; MR: mitral regurgitation; RBBB: right bundle branch block; LBBB: left bundle branch block; LAFB: left anterior fascicular block; ACE-I: angiotensin converting enzyme inhibitors; ARB: angiotensin II receptor blocker; CKD: chronic kidney disease; ESKD: end-stage kidney disease.

**Table 2:** Univariable regression logistic analysis for clinical characteristics associated with advanced IAB.

	Univariable Analysis	
	OR (95% CI)	*p*
Age	0.99 (0.96–1.03)	0.74
Male gender	0.76 (3.1–1.8)	0.54
Creatinine	1.18 (0.91–1.53)	0.22
Diabetes Mellitus	1.36 (0.53–3.49)	0.52
Hypertension	1.90 (0.73–4.92)	0.19
CAD	0.83 (0.26–2.64)	0.75
AF	0.51(0.43–0.59)	0.99
LA diameter	1.12 (0.81–1.53)	0.09
LVEF	1.00 (0.84–1.19)	0.99
Mild MR	0.45 (0.03–5.84)	0.53
Mild AR	1.87 (0.13–26.3)	0.65
1st-degree AV block	0.48 (0.06–3.94)	0.46
RBBB	1.98 (0.49–7.96)	0.33
LBBB	1.01 (0.43–0.60)	0.99
LAFB	2.24 (0.64–7.76)	0.20
GFR > 60 mL/min/1.73 m^2^	0.20 (0.06–0.72)	0.01
GFR < 60 mL/min/1.73 m^2^	1.31 (0.51–3.36)	0.57
ESKD	2.68 (1.09–6.62)	0.03
Oral Anticoagulant Drugs	1.36 (0.53–3.49)	0.52
ACE-I/ARB	1.33 (0.54–3.26)	0.54
β-blockers	0.53 (0.18–1.52)	0.22
Antiarrhythmic drugs	3.05 (0.70–13.2)	0.16

CAD: coronary artery disease; AF: atrial fibrillation; LA: left atrial; LVEF: left ventricular ejection fraction; AR: aortic regurgitation; MR: mitral regurgitation; RBBB: right bundle branch block; LBBB: left bundle branch block; LAFB: left anterior fascicular block; GFR: glomerular filtration rate; ESKD: end-stage kidney disease; ACE-I: angiotensin-converting enzyme inhibitors; ARB: angiotensin II receptor blocker.

## Data Availability

The data presented in this study are available on request from the corresponding author.
